# Crystal structure of [Y_6_(μ_6_-O)(μ_3_-OH)_8_(H_2_O)_24_]I_8_·8H_2_O

**DOI:** 10.1107/S1600536814025434

**Published:** 2014-11-26

**Authors:** François Le Natur, Guillaume Calvez, Olivier Guillou, Carole Daiguebonne, Kevin Bernot

**Affiliations:** a INSA, UMR 6226, Institut des Sciences Chimiques de Rennes, 35 708 Rennes, France

**Keywords:** crystal structure, hexa­nuclear compounds, lanthanide compound, three-dimensional hydrogen-bonded network

## Abstract

The crystal structure of the title compound {systematic name: octa-μ_3_-hydroxido-μ_6_-oxido-hexa­kis­[tetra­aqua­yttrium(III)] octa­iodide octa­hydrate}, is characterized by the presence of the centrosymmetric mol­ecular entity [Y_6_(μ_6_-O)(μ_3_-OH)_8_(H_2_O)_24_]^8+^, in which the six Y^3+^ cations are arranged octa­hedrally around a μ_6_-O atom at the centre of the cationic complex. Each of the eight faces of the Y_6_ octa­hedron is capped by an μ_3_-OH group in the form of a distorted cube. In the hexa­nuclear entity, the Y^3+^ cations are coordinated by the central μ_6_-O atom, the O atoms of four μ_3_-OH and of four water mol­ecules. The resulting coordination sphere of the metal ions is a capped square-anti­prism. The crystal packing is quite similar to that of the ortho­rhom­bic [*Ln*_6_(μ_6_-O)(μ_3_-OH)_8_(H_2_O)_24_]I_8_·8H_2_O structures with *Ln* = La—Nd, Eu—Tb, Dy, except that the title compound exhibits a slight monoclinic distortion. The proximity of the cationic complexes and the lattice water mol­ecules leads to the formation of a three-dimensional hydrogen-bonded network of medium strength.

## Abstract 2

In the crystal structure of a hexa­nuclear Y^3+^ compound, the six Y^3+^ cations are arranged octa­hedrally around an μ_6_-O atom at the centre of the cationic complex. Each of the eight faces of the Y_6_ octa­hedron is capped by an μ_3_-OH group in the form of a distorted cube. The proximity of the cationic complexes and lattice water mol­ecules leads to the formation of a three-dimensional hydrogen-bonded network of medium strength.

## Chemical context   

Rare-earth-based oxido-hydroxido polynuclear complexes are of inter­est because of their unique luminescence (Chen *et al.*, 2010 ▶; Le Natur *et al.*, 2013 ▶; Petit *et al.*, 2009 ▶), magnetic properties (Abbas *et al.*, 2010 ▶; Xu *et al.*, 2011 ▶) or structural characteristics (Zheng, 2001 ▶; Andrews *et al.*, 2013 ▶). Actually, in this kind of complex, the spatial proximity between metal ions affords cooperative/synergetic effects or energy-transfer mechanisms workable in terms of optical properties. For more than a decade, our group has been involved in the synthesis and the characterization of such rare-earth-based hexa­nuclear complexes (Calvez *et al.*, 2010 ▶). The hexa­nuclear complexes crystallize in different structures depending on the counter-anion (*e.g.* nitrate, perchlorate, iodide: Zak *et al.*, 1994 ▶; Wang *et al.*, 2000 ▶; Mudring *et al.*, 2006 ▶), the number of lattice water mol­ecules and/or the radius of the involved lanthanide ion. Since the pioneering work of Zak *et al.* (1994 ▶), we have developed a systematic synthetic procedure for the nitrate counter-anion complex with most of the rare earth elements (Calvez *et al.*, 2008 ▶, 2010 ▶). In this context, we have undertaken the study of a series of complexes based on the iodide counter-anion which have never been obtained with heavier rare earth ions. We report here the synthesis and crystal structure of the yttrium derivative.

## Structural commentary   

**Figure 1:**
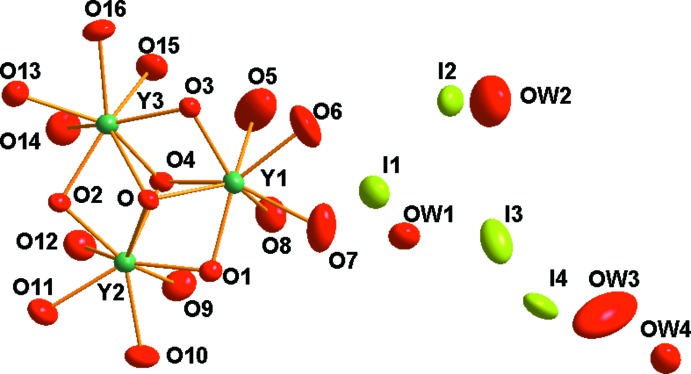
The asymmetric unit of the title complex. Displacement ellipsoids are drawn at the 50% probability level.

In contrast to the ortho­rhom­bic [*Ln*_6_(μ_6_-O)(μ_3_-OH)_8_(H_2_O)_24_]I_8_·8H_2_O structures with *Ln* = La—Nd, Eu—Tb, Dy (Mudring & Babai, 2005 ▶; Mudring *et al.*, 2006 ▶; Rukk *et al.*, 2009 ▶), the crystal structure of the yttrium member of this series has monoclinic symmetry, with the monoclinic angle close to 90° (Table 2 ▶). The asymmetric unit of [Y_6_(μ_6_-O)(μ_3_-OH)_8_(H_2_O)_24_]I_8_·8H_2_O contains half of the formula unit because the complete complex is situated on a centre of inversion. Three independent yttrium cations (Y1, Y2 and Y3), four oxygen atoms from μ_3_-hydroxyl groups (O1, O2, O3, O4), twelve oxygen atoms of terminal aqua ligands coordin­ating to each yttrium cation (Y1: O5, O6, O7, O8; Y2: O9, O10, O11, O12; Y3: O13, O14, O15 O16), one μ_6_-bridging O atom (O) lying on an inversion centre, four iodide anions (I1, I2, I3, I4) and four oxygen atoms of lattice water mol­ecules (O*W*1, O*W*2, O*W*3, O*W*4) are present in the crystal structure (Fig. 1 ▶). Calculations with the *SHAPE* software suite (Alvarez *et al.*, 2005 ▶) indicate that each of the coordination polyhedra surrounding the Y^3+^ ions is best described as a spherical capped square-anti­prism (Ruiz-Martínez *et al.*, 2010 ▶) with idealized *C*_4*v*_ symmetry. However, the true symmetry of this structural fragment in the title structure is *C*_1_.

**Figure 2:**
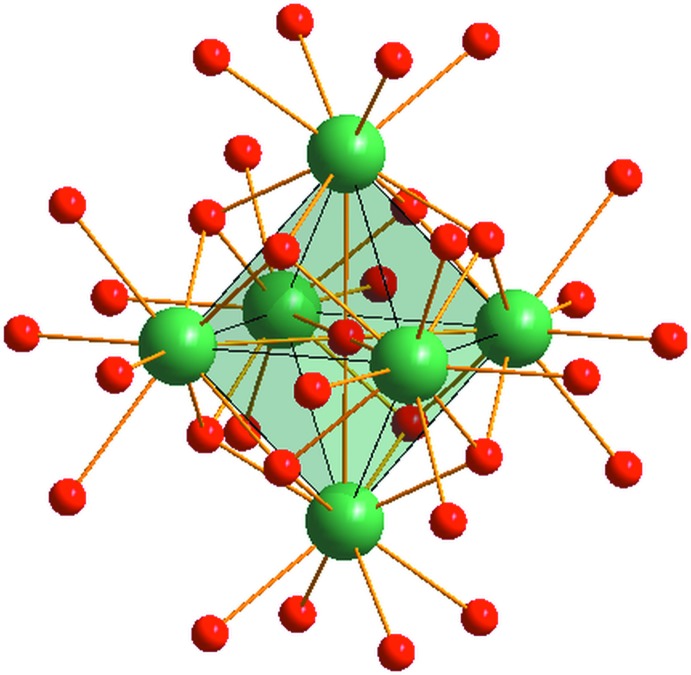
The OY_6_ octa­hedron in the complex [Y_6_(μ_6_-O)(μ_3_-OH)_8_(H_2_O)_24_]^8+^ cation. Y atoms are green and O atoms are red.

Since the μ_6_-O atom is located on an inversion centre and binds to six Y^3+^ cations, a slightly distorted anion-centred [OY_6_] octa­hedron results (Fig. 2 ▶). The average of the Y⋯Y distances between adjacent cations in the octa­hedron is found to be 3.536 Å. The mean Y—(μ_6_-O) distance is 2.537 Å, while the averaged Y—(μ_3_-OH) is 2.34 Å. The hydroxide ions are situated above the eight faces of the OY_6_ octa­hedron and form a distorted cube around the octa­hedron (Fig. 2 ▶).

## Supra­molecular features   

**Figure 3:**
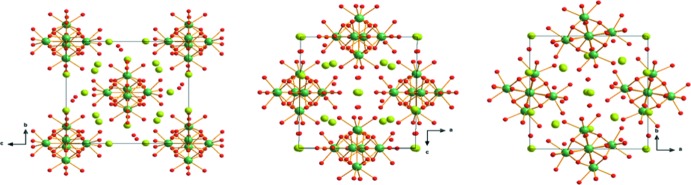
The crystal structure of [Y_6_(μ_6_-O)(μ_3_-OH)_8_(H_2_O)_24_]I_8_·8H_2_O in projections along [100], [010] and [001], respectively, from left to right. Y atoms are green, O atoms are red and I atoms are yellow.

The hexa­nuclear [Y_6_(μ_6_-O)(μ_3_-OH)_8_(H_2_O)_24_]^8+^ units are arranged in a body-centred fashion in the crystal structure. Each of these units is surrounded by twelve iodide anions, connecting the units to each other through Coulombic inter­actions. Although the hydrogen atoms of the water mol­ecules and hydroxide groups could not be located, the range of O⋯O distances between the cationic complex and the lattice water mol­ecules suggest the formation of medium-strength hydrogen bonds (Table 1 ▶). These inter­actions lead to the formation of a three-dimensional network in the structure (Fig. 3 ▶).

## Synthesis and crystallization   

Yttrium oxide Y_2_O_3_ (2 g, Strem Chemicals 4M) was dissolved in fresh hydro­iodic acid (9 ml, 57wt%, unstabilized from Acros Organics) under gentle heating (323 K). If the acid used is not fresh, it should be distilled twice. The clear solution was exposed to air under isothermal conditions (6 weeks). At this stage, the pH of the solution remains acidic. Large pale-yellow polyhedral crystals were separated manually from the solution and were mounted into a glass capillary.

## Refinement   

Crystal data, data collection and structure refinement details are summarized in Table 2 ▶. The hydrogen atoms from the water mol­ecules or hydroxide could not be assigned reliably and thus were not included in the refinement. However, they were taken into account for the chemical formula sum, moiety, weight, as well as for the absorption coefficient and the number of electrons in the unit cell.

## Supplementary Material

Crystal structure: contains datablock(s) I. DOI: 10.1107/S1600536814025434/wm5083sup1.cif

Structure factors: contains datablock(s) I. DOI: 10.1107/S1600536814025434/wm5083Isup2.hkl

CCDC reference: 1035218

Additional supporting information:  crystallographic information; 3D view; checkCIF report

## Figures and Tables

**Table 1:** Hydrogen-bond geometry ()

*D* *A*	*D* *A*
O7O*W*2	2.646(4)
O10O*W*3	2.764(1)
O13O*W*4	2.803(8)
O15O*W*1	2.767(2)
O16O*W*4	2.836(2)
O16O*W*1	2.851(6)

**Table 2:** Experimental details

Crystal data	
Chemical formula	[Y_6_O(OH)_8_(H_2_O)_24_]I_8_8H_2_O
*M*_r_	2277.24
Crystal system, space group	Monoclinic, *P*2_1_/*n*
Temperature (K)	293
*a*, *b*, *c* ()	12.9099(2), 14.8050(2), 14.7933(3)
()	90.821(1)
*V* (^3^)	2827.17(8)
*Z*	2
Radiation type	Mo *K*
(mm^1^)	10.54
Crystal size (mm)	0.18 0.14 0.1

Data collection	
Diffractometer	Nonius KappaCCD
Absorption correction	Gaussian (Coppens *et al.*, 1965 ▶)
*T*_min_, *T*_max_	0.018, 0.091
No. of measured, independent and observed [*I* > 2(*I*)] reflections	35352, 6374, 5449
*R*_int_	0.124
(sin /)_max_ (^1^)	0.647

Refinement	
*R*[*F* ^2^ > 2(*F* ^2^)], *wR*(*F* ^2^), *S*	0.067, 0.178, 1.11
No. of reflections	6374
No. of parameters	251
	*w* = 1/[^2^(*F*_o_^2^) + (0.0487*P*)^2^ + 43.8859*P*] where *P* = (*F*_o_^2^ + 2*F*_c_^2^)/3
_max_, _min_ (e ^3^)	2.62, 1.83

Computer programs: *COLLECT* (Nonius, 1998 ▶), *EVALCCD* (Duisenberg *et al.*, 2003 ▶), *SHELXS97* and *SHELXL97* (Sheldrick, 2008 ▶), *DIAMOND* (Brandenburg, 2006 ▶) and *publCIF* (Westrip, 2010 ▶).

## supplementary crystallographic information

### Crystal data

**Table:**

[Y_6_O(OH)_8_(H_2_O)_24_]I_8_·8H_2_O	*F*(000) = 2116
*M**_r_* = 2277.24	*D*_x_ = 2.675 Mg m^−^^3^
Monoclinic, *P*2_1_/*n*	Mo *K*α radiation, λ = 0.71073 Å
Hall symbol: -P 2yn	Cell parameters from 62388 reflections
*a* = 12.9099 (2) Å	θ = 2.9–27.5°
*b* = 14.8050 (2) Å	µ = 10.54 mm^−^^1^
*c* = 14.7933 (3) Å	*T* = 293 K
β = 90.821 (1)°	Block, colorless
*V* = 2827.17 (8) Å^3^	0.18 × 0.14 × 0.1 mm
*Z* = 2	

### Data collection

**Table:**

Nonius KappaCCD diffractometer	6374 independent reflections
Radiation source: fine-focus sealed tube	5449 reflections with *I* > 2σ(*I*)
Graphite monochromator	*R*_int_ = 0.124
CCD rotation images, thick slices scans	θ_max_ = 27.4°, θ_min_ = 3.1°
Absorption correction: gaussian (Coppens *et al.*, 1965)	*h* = −16→16
*T*_min_ = 0.018, *T*_max_ = 0.091	*k* = −19→18
35352 measured reflections	*l* = −19→19

### Refinement

**Table:**

Refinement on *F*^2^	Primary atom site location: structure-invariant direct methods
Least-squares matrix: full	Secondary atom site location: difference Fourier map
*R*[*F*^2^ > 2σ(*F*^2^)] = 0.067	*w* = 1/[σ^2^(*F*_o_^2^) + (0.0487*P*)^2^ + 43.8859*P*] where *P* = (*F*_o_^2^ + 2*F*_c_^2^)/3
*wR*(*F*^2^) = 0.178	(Δ/σ)_max_ = 0.001
*S* = 1.11	Δρ_max_ = 2.62 e Å^−^^3^
6374 reflections	Δρ_min_ = −1.83 e Å^−^^3^
251 parameters	Extinction correction: *SHELXL97* (Sheldrick, 2008), Fc^*^=kFc[1+0.001xFc^2^λ^3^/sin(2θ)]^-1/4^
0 restraints	Extinction coefficient: 0.00258 (19)

### Special details

**Table:**

Experimental. 6336 sampling points
Geometry. All s.u.'s (except the s.u. in the dihedral angle between two l.s. planes) are estimated using the full covariance matrix. The cell s.u.'s are taken into account individually in the estimation of s.u.'s in distances, angles and torsion angles; correlations between s.u.'s in cell parameters are only used when they are defined by crystal symmetry. An approximate (isotropic) treatment of cell s.u.'s is used for estimating s.u.'s involving l.s. planes.
Refinement. Refinement of *F*^2^ against ALL reflections. The weighted *R*-factor *wR* and goodness of fit *S* are based on *F*^2^, conventional *R*-factors *R* are based on *F*, with *F* set to zero for negative *F*^2^. The threshold expression of *F*^2^ > 2σ(*F*^2^) is used only for calculating *R*-factors(gt) *etc*. and is not relevant to the choice of reflections for refinement. *R*-factors based on *F*^2^ are statistically about twice as large as those based on *F*, and *R*- factors based on ALL data will be even larger.

### Fractional atomic coordinates and isotropic or equivalent isotropic displacement parameters (Å^2^)

**Table:**

	*x*	*y*	*z*	*U*_iso_*/*U*_eq_	
Y1	0.50083 (7)	1.00150 (6)	1.16560 (6)	0.0335 (2)	
Y2	0.46090 (7)	1.16586 (6)	0.99794 (6)	0.0317 (2)	
Y3	0.30677 (7)	0.96613 (6)	0.99944 (6)	0.0323 (2)	
I1	0.49148 (9)	0.82346 (8)	0.49868 (6)	0.0707 (3)	
I2	0.28137 (7)	0.72379 (7)	0.24171 (7)	0.0650 (3)	
I3	0.48625 (14)	0.50289 (7)	0.14980 (8)	0.0932 (4)	
I4	0.78359 (8)	0.78678 (8)	0.26729 (8)	0.0792 (4)	
O	0.5000	1.0000	1.0000	0.0293 (17)	
O1	0.3633 (5)	1.0806 (4)	1.0999 (4)	0.0328 (13)	
O2	0.4097 (5)	0.8826 (4)	1.1002 (4)	0.0313 (13)	
O3	0.3622 (5)	1.0773 (4)	0.8978 (4)	0.0321 (13)	
O4	0.4081 (5)	0.8803 (4)	0.9008 (4)	0.0335 (13)	
O5	0.6563 (13)	1.0251 (17)	1.2721 (11)	0.150 (8)	
O6	0.5306 (14)	0.8859 (13)	1.2758 (10)	0.136 (7)	
O7	0.3511 (12)	0.9672 (11)	1.2711 (9)	0.106 (5)	
O8	0.4775 (9)	1.1210 (7)	1.2708 (6)	0.069 (3)	
O9	0.4402 (8)	1.2676 (6)	1.1305 (7)	0.064 (2)	
O10	0.2918 (7)	1.2392 (6)	0.9958 (7)	0.065 (2)	
O11	0.4402 (7)	1.2644 (6)	0.8649 (7)	0.060 (2)	
O12	0.5819 (8)	1.2981 (6)	0.9988 (7)	0.066 (2)	
O13	0.1968 (6)	0.9524 (7)	1.1339 (6)	0.058 (2)	
O14	0.2216 (8)	0.8163 (7)	1.0027 (8)	0.075 (3)	
O15	0.1950 (6)	0.9452 (7)	0.8664 (6)	0.059 (2)	
O16	0.1578 (7)	1.0700 (7)	0.9987 (7)	0.063 (2)	
OW1	−0.0079 (7)	0.8851 (8)	0.8689 (7)	0.070 (3)	
OW2	0.2083 (19)	0.5431 (17)	0.0762 (13)	0.164 (8)	
OW3	0.7261 (18)	0.5806 (14)	0.051 (2)	0.216 (13)	
OW4	0.9948 (7)	0.8828 (8)	0.1328 (7)	0.069 (3)	

### Atomic displacement parameters (Å^2^)

**Table:**

	*U*^11^	*U*^22^	*U*^33^	*U*^12^	*U*^13^	*U*^23^
Y1	0.0380 (5)	0.0307 (5)	0.0318 (4)	0.0014 (3)	−0.0006 (3)	0.0000 (3)
Y2	0.0313 (4)	0.0271 (4)	0.0367 (5)	−0.0001 (3)	−0.0010 (3)	0.0003 (3)
Y3	0.0297 (4)	0.0292 (4)	0.0381 (5)	−0.0001 (3)	−0.0011 (3)	0.0003 (3)
I1	0.0799 (7)	0.0740 (7)	0.0580 (5)	0.0010 (5)	−0.0028 (4)	−0.0014 (4)
I2	0.0624 (5)	0.0599 (5)	0.0731 (6)	−0.0069 (4)	0.0146 (4)	0.0202 (4)
I3	0.1624 (13)	0.0473 (5)	0.0701 (7)	−0.0080 (6)	0.0074 (7)	0.0003 (4)
I4	0.0647 (6)	0.0771 (7)	0.0950 (8)	0.0062 (5)	−0.0237 (5)	0.0377 (6)
O	0.035 (4)	0.021 (4)	0.032 (4)	0.001 (3)	−0.002 (3)	0.002 (3)
O1	0.032 (3)	0.028 (3)	0.039 (3)	−0.001 (2)	0.001 (3)	−0.003 (3)
O2	0.029 (3)	0.023 (3)	0.042 (3)	0.001 (2)	0.000 (3)	0.005 (3)
O3	0.029 (3)	0.033 (3)	0.034 (3)	−0.002 (2)	−0.003 (2)	0.003 (3)
O4	0.029 (3)	0.033 (3)	0.038 (3)	0.000 (2)	−0.006 (3)	0.000 (3)
O5	0.099 (11)	0.26 (3)	0.090 (11)	0.009 (13)	−0.001 (9)	−0.023 (13)
O6	0.157 (15)	0.171 (17)	0.080 (9)	0.027 (12)	0.002 (9)	0.065 (10)
O7	0.127 (11)	0.120 (11)	0.070 (7)	−0.043 (9)	0.026 (7)	−0.005 (7)
O8	0.101 (8)	0.057 (6)	0.050 (5)	0.002 (5)	0.000 (5)	−0.018 (4)
O9	0.064 (6)	0.051 (5)	0.078 (6)	−0.003 (4)	0.004 (5)	−0.020 (5)
O10	0.054 (5)	0.052 (5)	0.089 (7)	0.018 (4)	−0.004 (5)	0.006 (5)
O11	0.058 (5)	0.047 (5)	0.075 (6)	0.001 (4)	−0.006 (4)	0.020 (4)
O12	0.067 (6)	0.048 (5)	0.082 (7)	−0.007 (4)	−0.004 (5)	0.007 (5)
O13	0.041 (4)	0.073 (6)	0.061 (5)	0.000 (4)	0.005 (4)	0.010 (4)
O14	0.062 (6)	0.065 (6)	0.098 (8)	−0.027 (5)	−0.003 (5)	0.002 (6)
O15	0.045 (4)	0.075 (6)	0.058 (5)	−0.004 (4)	−0.018 (4)	−0.005 (4)
O16	0.041 (4)	0.079 (6)	0.071 (6)	0.023 (4)	0.002 (4)	−0.006 (5)
OW1	0.051 (5)	0.086 (7)	0.072 (6)	−0.006 (5)	−0.004 (4)	−0.009 (5)
OW2	0.19 (2)	0.19 (2)	0.114 (14)	0.012 (17)	0.028 (13)	−0.005 (14)
OW3	0.158 (19)	0.091 (13)	0.40 (4)	0.027 (13)	0.09 (2)	0.004 (19)
OW4	0.052 (5)	0.086 (7)	0.068 (6)	−0.010 (5)	−0.006 (4)	0.010 (5)

### Geometric parameters (Å, º)

**Table:**

Y1—O2	2.321 (6)	Y2—O11	2.462 (9)
Y1—O3^i^	2.326 (6)	Y2—O9	2.490 (9)
Y1—O1	2.328 (7)	Y2—O12	2.506 (9)
Y1—O4^i^	2.332 (7)	Y2—O	2.5070 (9)
Y1—O8	2.378 (9)	Y3—O2	2.336 (6)
Y1—O6	2.390 (14)	Y3—O3	2.347 (6)
Y1—O	2.4497 (9)	Y3—O4	2.348 (7)
Y1—O7	2.553 (13)	Y3—O1	2.362 (6)
Y1—O5	2.557 (18)	Y3—O15	2.443 (8)
Y2—O2^i^	2.341 (6)	Y3—O16	2.462 (8)
Y2—O3	2.341 (6)	Y3—O13	2.469 (8)
Y2—O4^i^	2.344 (6)	Y3—O14	2.477 (10)
Y2—O1	2.347 (6)	Y3—O	2.5444 (9)
Y2—O10	2.438 (9)		
			
O2—Y1—O3^i^	80.5 (2)	O10—Y2—O	128.0 (2)
O2—Y1—O1	80.1 (2)	O11—Y2—O	127.6 (2)
O3^i^—Y1—O1	131.5 (2)	O9—Y2—O	127.3 (3)
O2—Y1—O4^i^	130.4 (2)	O12—Y2—O	129.8 (2)
O3^i^—Y1—O4^i^	79.4 (2)	O2—Y3—O3	127.1 (2)
O1—Y1—O4^i^	80.4 (2)	O2—Y3—O4	78.1 (2)
O2—Y1—O8	140.2 (3)	O3—Y3—O4	78.7 (2)
O3^i^—Y1—O8	137.8 (3)	O2—Y3—O1	79.1 (2)
O1—Y1—O8	78.2 (3)	O3—Y3—O1	78.8 (2)
O4^i^—Y1—O8	77.7 (3)	O4—Y3—O1	127.5 (2)
O2—Y1—O6	79.4 (5)	O2—Y3—O15	140.4 (3)
O3^i^—Y1—O6	78.5 (5)	O3—Y3—O15	75.8 (3)
O1—Y1—O6	139.4 (5)	O4—Y3—O15	76.0 (3)
O4^i^—Y1—O6	138.4 (5)	O1—Y3—O15	140.5 (3)
O8—Y1—O6	96.2 (6)	O2—Y3—O16	140.4 (3)
O2—Y1—O	65.23 (16)	O3—Y3—O16	78.7 (3)
O3^i^—Y1—O	65.46 (16)	O4—Y3—O16	141.0 (3)
O1—Y1—O	66.07 (16)	O1—Y3—O16	77.8 (3)
O4^i^—Y1—O	65.21 (16)	O15—Y3—O16	67.9 (3)
O8—Y1—O	131.5 (3)	O2—Y3—O13	76.8 (3)
O6—Y1—O	132.4 (5)	O3—Y3—O13	139.3 (3)
O2—Y1—O7	73.7 (4)	O4—Y3—O13	142.0 (3)
O3^i^—Y1—O7	137.2 (4)	O1—Y3—O13	74.2 (3)
O1—Y1—O7	77.0 (4)	O15—Y3—O13	107.4 (3)
O4^i^—Y1—O7	142.9 (4)	O16—Y3—O13	66.2 (3)
O8—Y1—O7	69.2 (4)	O2—Y3—O14	76.3 (3)
O6—Y1—O7	63.7 (6)	O3—Y3—O14	141.2 (3)
O—Y1—O7	128.1 (3)	O4—Y3—O14	77.2 (3)
O2—Y1—O5	138.5 (6)	O1—Y3—O14	139.8 (3)
O3^i^—Y1—O5	73.9 (4)	O15—Y3—O14	69.2 (4)
O1—Y1—O5	140.9 (6)	O16—Y3—O14	102.3 (4)
O4^i^—Y1—O5	76.2 (5)	O13—Y3—O14	69.5 (4)
O8—Y1—O5	66.5 (5)	O2—Y3—O	63.48 (15)
O6—Y1—O5	64.0 (7)	O3—Y3—O	63.63 (15)
O—Y1—O5	127.5 (4)	O4—Y3—O	63.47 (15)
O7—Y1—O5	104.2 (5)	O1—Y3—O	64.06 (15)
O2^i^—Y2—O3	79.8 (2)	O15—Y3—O	126.5 (2)
O2^i^—Y2—O4^i^	78.0 (2)	O16—Y3—O	130.0 (3)
O3—Y2—O4^i^	128.4 (2)	O13—Y3—O	126.1 (2)
O2^i^—Y2—O1	128.9 (2)	O14—Y3—O	127.7 (3)
O3—Y2—O1	79.2 (2)	Y1^i^—O—Y1	180.0
O4^i^—Y2—O1	79.7 (2)	Y1^i^—O—Y2^i^	90.07 (3)
O2^i^—Y2—O10	140.8 (3)	Y1—O—Y2^i^	89.93 (3)
O3—Y2—O10	76.2 (3)	Y1^i^—O—Y2	89.93 (3)
O4^i^—Y2—O10	140.9 (3)	Y1—O—Y2	90.07 (3)
O1—Y2—O10	76.1 (3)	Y2^i^—O—Y2	180.0
O2^i^—Y2—O11	75.9 (3)	Y1^i^—O—Y3^i^	89.73 (3)
O3—Y2—O11	77.0 (3)	Y1—O—Y3^i^	90.27 (3)
O4^i^—Y2—O11	138.8 (3)	Y2^i^—O—Y3^i^	89.76 (3)
O1—Y2—O11	141.2 (3)	Y2—O—Y3^i^	90.24 (3)
O10—Y2—O11	68.8 (3)	Y1^i^—O—Y3	90.27 (3)
O2^i^—Y2—O9	139.4 (3)	Y1—O—Y3	89.73 (3)
O3—Y2—O9	140.6 (3)	Y2^i^—O—Y3	90.24 (3)
O4^i^—Y2—O9	76.0 (3)	Y2—O—Y3	89.76 (3)
O1—Y2—O9	75.8 (3)	Y3^i^—O—Y3	180.0
O10—Y2—O9	68.6 (3)	Y1—O1—Y2	97.2 (2)
O11—Y2—O9	105.1 (4)	Y1—O1—Y3	97.4 (2)
O2^i^—Y2—O12	78.0 (3)	Y2—O1—Y3	98.4 (2)
O3—Y2—O12	140.8 (3)	Y1—O2—Y3	98.3 (2)
O4^i^—Y2—O12	77.3 (3)	Y1—O2—Y2^i^	97.4 (2)
O1—Y2—O12	139.3 (3)	Y3—O2—Y2^i^	99.9 (2)
O10—Y2—O12	102.2 (3)	Y1^i^—O3—Y2	97.3 (2)
O11—Y2—O12	66.5 (3)	Y1^i^—O3—Y3	98.5 (2)
O9—Y2—O12	66.3 (3)	Y2—O3—Y3	99.0 (2)
O2^i^—Y2—O	64.02 (15)	Y1^i^—O4—Y2^i^	97.2 (2)
O3—Y2—O	64.31 (16)	Y1^i^—O4—Y3	98.3 (2)
O4^i^—Y2—O	64.12 (16)	Y2^i^—O4—Y3	99.4 (2)
O1—Y2—O	64.86 (16)		

Symmetry code: (i) −*x*+1, −*y*+2, −*z*+2.

### Hydrogen-bond geometry (Å)

**Table:**

*D*—H···*A*	*D*···*A*
O7···O*W*2	2.646 (4)
O10···O*W*3	2.764 (1)
O13···O*W*4	2.803 (8)
O15···O*W*1	2.767 (2)
O16···O*W*4	2.836 (2)
O16···O*W*1	2.851 (6)

## References

[bb1] Abbas, G., Lan, Y., Kostakis, G. E., Wernsdorfer, W., Anson, C. E. & Powell, A. K. (2010). *Inorg. Chem.* **49**, 8067–8072. 10.1021/ic1011605 20704320

[bb2] Alvarez, S., Alemany, P., Casanova, D., Cirera, J., Llunell, M. & Avnir, D. (2005). *Coord. Chem. Rev.* **249**, 1693–1708.

[bb3] Andrews, P. C., Gee, W. J., Junk, P. C. & Massi, M. (2013). *New J. Chem.* **37**, 35–48.

[bb4] Brandenburg, K. (2006). *DIAMOND*. Crystal Impact GbR, Bonn, Germany.

[bb5] Calvez, G., Daiguebonne, C., Guillou, O., Pott, T., Méléard, P. & Le Dret, F. (2010). *C. R. Chim.* **13**, 715–730.

[bb6] Calvez, G., Guillou, O., Daiguebonne, C., Car, P. E., Guillerm, V., Gérault, Y., Le Dret, F. & Mahé, N. (2008). *Inorg. Chim. Acta*, **361**, 2349–2356.

[bb7] Chen, X.-Y., Yang, X. & Holliday, B. J. (2010). *Inorg. Chem.* **49**, 2583–2585. 10.1021/ic902513z 20163123

[bb8] Coppens, P., Leiserowitz, L. & Rabinovich, D. (1965). *Acta Cryst.* **18**, 1035–1038.

[bb9] Duisenberg, A. J. M., Kroon-Batenburg, L. M. J. & Schreurs, A. M. M. (2003). *J. Appl. Cryst.* **36**, 220–229.

[bb10] Le Natur, F., Calvez, G., Daiguebonne, C., Guillou, G., Bernot, K., Ledoux, J., Le Pollès, L. & Roiland, C. (2013). *Inorg. Chem.* **52**, 6720–6730. 10.1021/ic4008697 23692502

[bb11] Mudring, A.-V. & Babai, A. (2005). *Z. Anorg. Allg. Chem.* **631**, 261–263.

[bb12] Mudring, A.-V., Timofte, T. & Babai, A. (2006). *Inorg. Chem.* **45**, 5162–5166. 10.1021/ic051234s 16780339

[bb13] Nonius (1998). *COLLECT*. Nonius BV, Delft, The Netherlands.

[bb14] Petit, S., Baril-Robert, F., Pilet, G., Reber, C. & Luneau, D. (2009). *Dalton Trans.* pp. 6809–6815. 10.1039/b822883c 19690693

[bb15] Ruiz-Martínez, A., Casanova, D. & Alvarez, S. (2010). *Chem. Eur. J.* **16**, 6567–6581. 10.1002/chem.200902996 20414909

[bb16] Rukk, N. S., Al’bov, D. V., Skryabina, A. Y., Osipov, R. A. & Alikberova, L. Y. (2009). *Russ. J. Coord. Chem.* **35**, 12–14.

[bb17] Sheldrick, G. M. (2008). *Acta Cryst.* A**64**, 112–122. 10.1107/S0108767307043930 18156677

[bb18] Wang, R., Carducci, M. D. & Zheng, Z. (2000). *Inorg. Chem.* **39**, 1836–1837. 10.1021/ic991391p 11428100

[bb19] Westrip, S. P. (2010). *J. Appl. Cryst.* **43**, 920–925.

[bb20] Xu, X., Zhao, L., Xu, G.-F., Guo, Y.-N., Tang, J. & Liu, Z. (2011). *Dalton Trans.* **40**, 6440–6444. 10.1039/c1dt10450k 21566804

[bb21] Zak, Z., Unfried, P. & Giester, G. (1994). *J. Alloys Compd*, **205**, 235–242.

[bb22] Zheng, Z. (2001). *Chem. Commun.* pp. 2521–2529.

